# Acute Profound Thrombocytopenia Induced by Eptifibatide Causing Diffuse Alveolar Hemorrhage

**DOI:** 10.1155/2021/8817067

**Published:** 2021-07-15

**Authors:** Gregory Byrd, Sabina Custovic, David Byrd, Deanna Ingrassia Miano, Jasdeep Bathla, Antonious Attallah

**Affiliations:** ^1^Department of Internal Medicine, Ascension Macomb-Oakland Hospital, 11800 12 Mile Road, Warren, MI 48093, USA; ^2^Michigan State University College of Osteopathic Medicine, 965 Wilson Road, East Lansing, MI 48824, USA

## Abstract

**Background:**

Eptifibatide is a glycoprotein IIb/IIIa (GP IIb/IIIa) receptor inhibitor which prevents platelet activation. The mechanism in which eptifibatide causes profound thrombocytopenia is poorly understood. One hypothesis suggests antibody-dependent pathways which cause thrombocytopenia upon subsequent reexposure to eptifibatide. This case reports acute profound thrombocytopenia (platelets < 20 × 10^3^/mm^3^) within 24 hours of administration. Alveolar hemorrhage occurred during a second eptifibatide infusion 5 days after initial asymptomatic eptifibatide treatment. *Case Presentation*. A 50-year-old male presenting with a STEMI was treated with eptifibatide during cardiac catheterization. Twelve hours posttreatment, the patient encountered profound thrombocytopenia and hemoptysis. The patient was briefly intubated for airway protection. The patient was stabilized after receiving platelet transfusion and fully recovered.

**Conclusion:**

This is one of several cases reported on eptifibatide causing acute profound thrombocytopenia and subsequent alveolar hemorrhage. This case supports the theory in which antibodies contribute to eptifibatide-induced thrombocytopenia.

## 1. Introduction

Eptifibatide is a glycoprotein IIb/IIIa receptor inhibitor which works to prevent cross-linkage and platelet plug formation. Eptifibatide is used as standard antithrombotic therapy in the management of acute coronary syndrome (ACS) with unstable angina (UA) or non-ST elevation myocardial infarctions (NSTEMI), as well as in those undergoing percutaneous coronary intervention (PCI). Despite clinical efficacy, acute profound thrombocytopenia within 24 hours of treatment may develop with eptifibatide. Diffuse alveolar hemorrhage (DAH), another rare complication of GP IIb/IIIa inhibitor therapy, is diagnosed using typical symptoms of dyspnea and hemoptysis in addition to radiographic changes [1, 2]. Here, we present a case in which eptifibatide causes thrombocytopenia and alveolar hemorrhage upon second exposure.

## 2. Case Presentation

**Figure 1:**
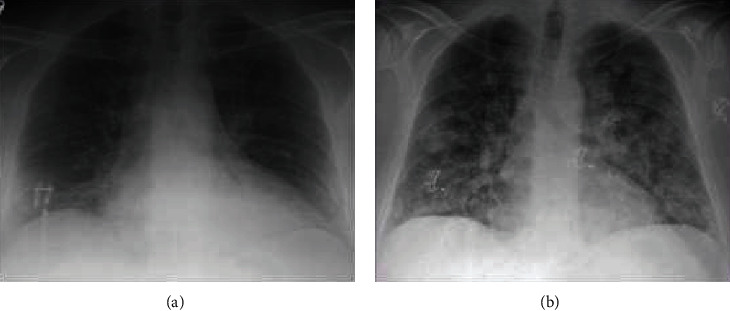
(a) Approximately 24 hours prior to the development of diffuse pulmonary hemorrhage. (b) After the development of severe cough and hemoptysis which required airway protection through intubation.

This case outlines a 50-year-old male with past medical history significant for diabetes, hypertension, and coronary artery disease (CAD) who presented to the hospital with chest pain. Five days prior, the patient underwent cardiac catheterization with intervention; 3 stents were placed in the left anterior descending artery. Upon presentation, the patient was found to have an inferior-posterior STEMI. He received multiple balloon angioplasties to a 100% occluded first obtuse marginal artery with in-stent thrombosis. At this time, the patient was on aspirin and Ticagrelor. He was further treated with eptifibatide during the catheterization; a double bolus was given during the case and was to be continued for 12 hours thereafter. The patient developed a severe cough and mild-moderate hemoptysis. In subsequent chest X-rays, diffuse opacities were seen, and the patient was eventually intubated for airway protection (Figure 1). The patient's platelets decreased from 370k to 5k within 12 hours. It is worth noting that the patient was treated with eptifibatide during his initial catheterization 5 days prior as well, with no complications. Due to his acute profound thrombocytopenia and persistent hemoptysis, the patient received 1 unit of platelets; the Ticagrelor and eptifibatide were discontinued. Soon after, his platelets began to rebound. In addition, the patient was extubated the following morning. The patient did not require further intervention, made full recovery, and was discharged 9 days later.

## 3. Discussion

**Figure 2:**
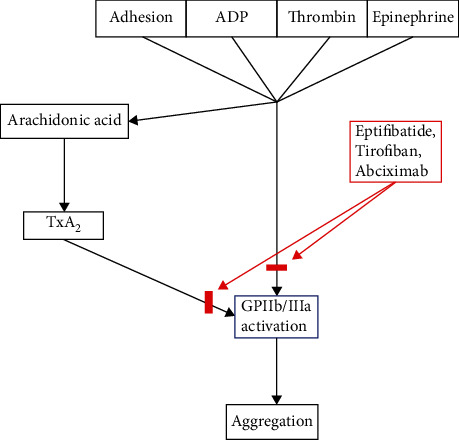
Flow chart demonstrating platelet activation via GP IIb/IIIa activation. Eptifibatide and similar drugs (red) inhibit the aggregation pathway by blocking GP IIb/IIIa functionality.

Today, antiplatelet use is standard in patients with ACS undergoing cardiac catheterization. Eptifibatide works by reversibly inhibiting platelet activation by binding to GP IIb/IIIa receptors on the surface of the platelets. The GP IIb/IIIa receptor is an integrin receptor found exclusively on the surface of the platelets. These receptors respond to components such as von Willebrand factor (vWF), fibronectin, and fibrinogen, which in turn activate clotting [3, 4]. After the receptor is activated, it leads to cross-linking of platelets causing aggregation and thus the formation of a thrombus. Eptifibatide acts as a ligand mimetic; it adheres to the GP IIb/IIIa recognition site and blocks vWF, fibronectin, and fibrinogen from binding, ultimately inhibiting platelet aggregation [5]. The GP IIb/IIIa receptor is especially interesting for several reasons. One reason is that it appears to be hidden when the platelet is not activated [3]. Additionally, there is a staggering amount of the receptors on each platelet, an estimated 50,000 receptors/cell, giving platelets a powerful ability to aggregate and form a clot [3]. This is likely the reason why GP IIb/IIIa inhibitors are very successful at inhibiting platelet aggregation (Figure 2).

Drug-induced thrombocytopenia is a known complication of GP IIb/IIIa inhibitors [6, 7]. Acute profound thrombocytopenia, commonly defined as platelets < 20 × 10^3^/mm^3^ within 24 hours of administration, is a rare complication of eptifibatide [8]. In our patient's case, a drop of platelets from 370k to 5k within 12 hours of eptifibatide reexposure, as well as platelet rebound after eptifibatide discontinuation, leads us to believe that this was an instance of drug-induced thrombocytopenia. Drug-induced thrombocytopenia can be confirmed with serum eptifibatide-dependent platelet-reactive antibody testing; however, these labs were not conducted in this particular case [9].

The estimated occurrence of thrombocytopenia with eptifibatide use is approximately 3%, and acute profound thrombocytopenia occurs around 0.1-1% [4]. According to a database published by the University of Oklahoma which analyzed cases of eptifibatide-induced thrombocytopenia, only 16 of 34 reported cases had bleeding associated with profound thrombocytopenia. The database specifically noted that 10 of the 16 cases had major bleeding (i.e., melena, gross hematuria, and excessive epistaxis). Six of the 16 cases had minor bleeding or trivial bleeding (i.e., petechiae and purpura) [10].

The mechanism of how eptifibatide causes thrombocytopenia is currently unknown. Mechanisms involving both antibody-dependent and antibody-independent pathways have been proposed. One review found several independent risk factors for thrombocytopenia, including age > 65, low BMI, and a baseline platelet count of <180k [11]. The fact that thrombocytopenia can occur after the first dose of eptifibatide supports current theories of an antibody-independent pathway. However, there is some data to suggest that there are either naturally occurring antibodies against the ligand binding sites where these antiplatelet molecules act [12, 13]. One review found that 5 out of 5 patients who developed thrombocytopenia had high IgG titers with cross-reactivity to GP IIb/IIIa, while 1 of the 5 patients had high IgG levels prior to eptifibatide administration. This data is suggestive of both mechanisms playing a role [9].

Eptifibatide-induced thrombocytopenia is a clinical diagnosis. The clinical picture of eptifibatide-induced thrombocytopenia appears as a rapidly dropping platelet count that is quickly reversed after discontinuation of the drug. The most important differentials to rule out are heparin-induced thrombocytopenia and pseudothrombocytopenia. Most other disorders are easily excluded due to a normal platelet count prior to the administration of the drug. In addition, typically, platelet counts only drop below 20k in eptifibatide-induced thrombocytopenia; other diagnoses rarely dip below this threshold.

Adequate treatment of eptifibatide-induced thrombocytopenia typically only requires removal of the offending agent, and platelets usually recover to near-normal levels within several days [12]. If profound thrombocytopenia is present or clinically significant bleeding occurs, both of which were seen in this patient, platelets may be transfused [12].

## 4. Conclusion

This case highlights another example of eptifibatide-induced thrombocytopenia and diffuse alveolar hemorrhage. Furthermore, this case may give more weight to the theory that drug-induced thrombocytopenia is driven by antibodies. Although this case does not prove this theory, it is consistent with the theory, as our patient did not have any adverse effects with his first exposure to eptifibatide.
